# Recurrent Unilateral E. coli Breast Infection in a Non-Lactating Women: A Rare Case Report

**DOI:** 10.7759/cureus.53675

**Published:** 2024-02-05

**Authors:** Vigneshkumar Palanisamy, Palan Thirunavukkarasu, Ruwan Wijesuriya

**Affiliations:** 1 General Surgery, St. John of God Midland Public and Private Hospital, Perth, AUS

**Keywords:** e. coli, antibiotics, unknown etiology, non lactating women, recurrent breast abscess

## Abstract

Breast abscess is a common infection of the breast in humans, particularly affecting females who are lactating. Lactation mastitis is present in 2%-3% of women and approximately 5%-11% of patients may develop abscess. However, breast abscess in non-lactating women is extremely rare and there has only been limited literature published on this. *Escherichia coli* (*E. coli*)* *is usually found in the gastrointestinal and urogenital system, with no previous documentation of an *E. coli* infection in the breast. This case report summarizes the rare case of a fit and healthy adult female healthcare worker who presented with a recurrence of a unilateral* E. coli *breast abscess within three years. On review, there have not been any similar documented cases.

## Introduction

Breast abscess is a common presentation to the hospital among lactating women. It is important to identify and treat promptly in order to prevent sepsis. Common management of breast abscesses is medical management with antibiotics (either intravenous or oral) or surgical management with incision and drainage. It is uncommon to see breast abscesses in non-lactating women [1], especially without any predisposing risk factors such as smoking, diabetes, or immunocompromised status. There has been only a limited number of cases reported in the literature regarding breast abscesses in non-lactating women and the usual microorganisms that cause breast abscesses are predominantly found on skin surfaces such as *Staphylococcus aureus*, Bacteroides spp, anaerobic streptococci, and *Pseudomonas aeruginosa* [2]. *Escherichia coli* (*E. coli*) is predominantly found in the gastrointestinal tract [3], making it unusual for *E. coli* to be found in the breast. This case report looks at one such patient who presented with recurrent episodes of *E. coli* breast abscess on the same breast within an interval of three years. Furthermore, there has been no previously documented evidence of a recurrent *E. coli* breast infection found in the literature review. An informed consent was obtained from the patient and hospital to document this case report.

## Case presentation

A 41-year-old female presented to our emergency department with primary complaints of pain and swelling of her left breast for the last five days, which has been gradually getting worse. This was associated with subjective fevers, rigors, and fatigue. She denied any discharge from the nipple. She reported feeling unwell preceding the development of this abscess. Her past medical history includes previous simple cyst with fibrocystic changes on USS at 9 o’clock identified in 2018. She developed an abscess in the same breast in 2021 that was aspirated, and the culture grew *E. coli*. She was adequately treated with antibiotics during this period. She recovered well and had no issues for the past two years. No past medical history of recurrent urinary tract infections (UTI) and no history of bowel diseases.

Furthermore, she also had two pregnancies and two live-born children. She has no significant family history of breast cancer or any other immunosuppressive or autoimmune diseases. She is a non-smoker, drinks socially (a few standard drinks a week), and does not use illicit drugs.

On examination, she appeared well from the bedside and did not appear septic. The vitals were stable and afebrile. On examining the breast, there was erythema, induration, and palpable lump present on the left breast of 3x4x2cm in the right upper quadrant 3cm from the nipple. The area was tender and warm to the touch. The nipple appeared normal with no active discharge. There was no axillary or cervical lymphadenopathy present.

**Figure 1:**
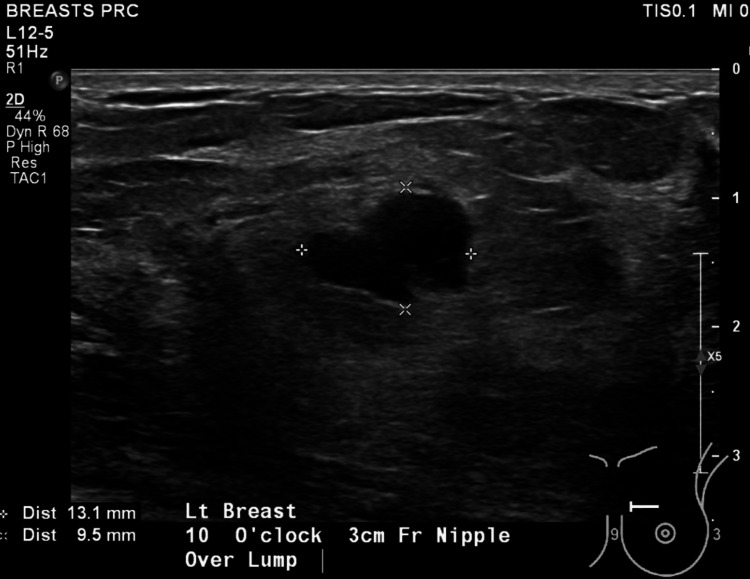
Ultrasound of her right breast on her second presentation.

The inflammatory markers were elevated with WCC: 13.0 and CRP: 56.4. Further investigation with breast ultrasonography (Figure 1) showed a lobulated cystic lesion and marginally thickened wall measuring 13x10x10 mm.

Given the size of the abscess was small, the decision was made to perform ultrasound-guided drainage rather than a surgical incision and drainage of the abscess. An ultrasound-guided aspiration was performed under aseptic conditions and 3mL of blood-stained pus was aspirated, which was sent for culture testing.

She was commenced on intravenous Piperacillin and Tazobactam antibiotic, which was sensitive to the previous *E. coli* culture sensitivity done in 2021. The pus culture and sensitivity results were obtained and had heavy growth of *E. coli*, which was pan-sensitive to all the common antibiotics. After discussion with the infectious diseases team, she was stepped down to oral Cephalexin for 14 days on discharge subsequently seen in the outpatient clinic and had investigations to identify the cause of *E. coli* breast abscess. These included blood tests and urine tests which turned out to be normal. Given all the tests to identify the source of *E. coli* infection were negative, the decision was made to rule out a colonic origin and distant spread. Hence, the colonoscopy was performed and returned negative with no abnormality.

The follow-up blood after four weeks showed no elevated inflammatory markers. She recovered well with no further complications. Given there were no positive findings in colonoscopy and there was no evidence of previous *E. coli* UTI, the etiology of the recurrent infection remains unknown.

## Discussion

Breast abscesses are usually divided into various categories. These include lactational (puerperal) and non-lactational (non-puerperal), and anatomically can be further divided into sub-areolar (within 1cm from the areolar) and non-sub-areolar (more than 1cm from the areolar).

The pathophysiology of breast abscess involves the obstruction or blockage of the lactiferous sinuses, which are reservoirs for milk during the postpartum period for women. This leads to the formation of abscesses. Causes of breast abscess in non-lactating women include trauma, granulomatous mastitis, and immunosuppressive diseases such as diabetes or rheumatoid arthritis [1].

The common organism that causes breast abscesses is *S. aureus*. However, there have been cases where Streptococci was detected [4]. Furthermore, there has been a case reported to have Mycobacterium chelonae in a 22-year-old Japanese woman, who was treated with incision and drainage and multiple antibacterial agents [5]. The origin of some atypical bacteria that cause breast abscesses remains unknown.

A literature review showed there was a case published by Simsek [6] that discussed a 36-year-old female who developed an *E. coli* breast abscess bilaterally. This was managed with surgical incision and drainage, and adequate antibiotics cover post drainage. A second case report published by Kokhreidze [7] discussed that there was a young female (29 years old) who had a unilateral breast abscess and was treated conservatively with antibiotics likely due to the relatively small size of the abscess.

As established, breast abscesses in non-lactating women are quite rare and there are limited cases reported. In our case, there are a few hypotheses, that could have led to the formation of recurrent breast abscess.

Firstly, she had her initial presentation in 2021. This was managed with intravenous and oral antibiotics. One hypothesis could be the possibility of bacteria being dormant following the treatment and not completely treated. Incorvaia summarized that bacterium can go to any extent to cope with changes in the environment (pH and temperature) [8]. One way is building a “fortress-like shell around their DNA,” thereby not having any way to detect them. Bacteria develop spores to protect themselves and stay inactive for years. They become active and form active infection when the host undergoes an immunosuppressive stage. Harvard University is currently in the process of identifying a new kind of cellular sensor, which detects the presence of nutrients [9] that could reactivate the organism. Based on this theory, it is possible that the same *E. coli* dormant bacteria could have been reactivated in our patient after two years. In the future, with the development of this cellular sensor, we will be able to detect and successfully eradicate to prevent the recurrence and formation of resistant organisms. This theory could be the crucial reason why our patient developed recurrent *E. coli* abscess in the same breast.

Secondly, our patient was working as a full-time healthcare worker. In a study by Kilic [10], they found that nurses were exposed to 73.8% of bacteria, 67.3% of viruses, and 55.5% of fungi in their work environment. Our patient had known fibrocystic changes in her breast and exposure to *E. coli* from a contact of a patient in her work environment could have led to the formation of abscess. Cook [11] stated that fibrocystic changes in the breast are associated with tenderness and swelling and can be a gateway for infection. Hence, to prevent this, healthcare workers should take all the preventative measures and practice good hand hygiene.

Furthermore, the management of these recurrent abscess should be discussed with the patient in detail. The traditional method of treatment has always been incision and drainage of the abscess [12]. However, Kataria summarized that repeated incision and drainage lead to prolonged healing time, difficulty with breastfeeding, and even reported cases of fistula formation. There have been multiple studies, which has identified that conservative management with antibiotics can be less invasive. Secondly, aspiration of the pus is also more preferred compared to open surgery [7].

As detailed above, although the first line of management is aspiration and antibiotics, surgical management should be considered in our patient if she presents with recurrent abscesses. There is a high probability of this patient representing a recurrence. Careful analysis of the abscess should be done with ultrasonography or MRI to identify the size and nature of the abscess. A multidisciplinary team approach is crucial and the risks and benefits of this should be discussed with our patient in the future if she is to present with another breast abscess.

## Conclusions

In conclusion, recurrent *E. coli* breast abscesses are quite rare among non-lactating women. All the other sources of *E. coli* infection in the body should be ruled out (urine, feces) at the time of presentation. Evidence has shown that treatment of the breast abscess is best managed by intravenous antibiotics and radiologically guided aspiration in comparison to surgical incision and drainage. There are many hypotheses explored in this case report. It is important to have ongoing research on the prevalence of dormant bacteria in such cases to identify their origin and prevent their occurrence.
